# Cardiac magnetic resonance imaging and gadolinium angiography for neonates and small infants: a 10-year single institutional experience

**DOI:** 10.1186/1532-429X-14-S1-O57

**Published:** 2012-02-01

**Authors:** Sheela Rangamani, Joby Varghese, James M Hammel, Scott E Fletcher, Kim F Duncan, David A Danford, Shelby Kutty

**Affiliations:** 1 University of Nebraska/Creighton University, Joint Division of Pediatric Cardiology, Children's Hospital and Medical Center, Omaha, NE, USA; 2 Division of Cardiovascular Surgery, University of Nebraska/Creighton University, Children's Hospital and Medical Center, Omaha, NE, USA; 3 Division of Anesthesia, Children's Hospital and Medical Center, Omaha, NE, USA

## Background

With increasing applications of cardiac magnetic resonance (CMR) for definitive diagnosis of congenital heart disease (CHD) in infants, safety of this technology is of particular interest in this age group. We report our ten-year experience with CMR in neonates and small infants with particular focus on the safety profile and incidence of adverse events.

## Methods

Clinical, anesthesia and nursing records of all patients (pts)<120 days of age who underwent CMR were reviewed. Variables including cardiac diagnosis, study duration, anesthesia type and agents used, prostaglandin E1 (PGE1) dependence and gadolinium (Gd) use were recorded. Serially recorded temperature, systemic saturation, and cardiac rhythm were analyzed. Primary outcome measure was any adverse event (AE) during or <24 hours after the procedure, including minor AE such as hypothermia (axillary temperature ≤95 F), desaturation (SpO2 drop ≥ 10% below baseline) and bradycardia (heart rate ≤100/ min). Secondary outcome measure was unplanned overnight hospitalization of out-pts.

## Results

Pts (n=143; 74 male, 69 female) had a median age of 7 days (1-117), and 98 were ≤30 days at the time of CMR. The median weight was 3.4 kg (1.4-6) and body surface area 0.22 m2 (0.13-0.32). There were 118 (83%) in-pts (108 receiving intensive care) and 25 (17%) out-pts. Indications were assessment of aortic arch (n=57), complex CHD (n=41), pulmonary veins (n=15), vascular ring (n=8), intracardiac mass (n=8), pulmonary artery (n=7), ventricular volume (n=4), and systemic veins (n=3). CMR was performed using a 1.5-T scanner (Philips), and a commercially available coil. CMR utilized general anesthesia (GA) in 86 pts, deep sedation in 50 and no sedation in 7. Gd angiography was performed in 136 pts. Fifty-nine pts were PGE1 dependent and 39 had a univentricular circulation. Among pts on PGE1, 43 (74%) received GA and 10 (26%) had deep sedation. Twelve pts (8%) had AE, 1 major and 11 minor. Nine of the12 pts had GA, and 3 had deep sedation. The single major AE was respiratory arrest after chloral hydrate sedation in a neonate (resuscitated without sequelae). Minor AE included desaturations (n=2), hypothermia (n=5), bradycardia (n=2), and bradycardia with hypoxemia (n=2). Incidence of minor AE was 9% for in-pts (vs. 4% for out-pts), and 8% for neonates (vs. 9% in pts ≥ 30 days). Incidence of minor AE was similar between PGE1 dependent infants and non-PGE1 group. There were no AE related to Gd angiography. Of 25 out-pts, 5 (20%) were admitted for overnight observation due to desaturations.

## Conclusions

CMR imaging can be accomplished safely in neonates and infants ≤ 120 days for a wide range of pre surgical cardiac indications. Incidence of AE was unrelated to pt age, complexity of heart disease, type of anesthesia and PGE1 dependence.

## Funding

None.

